# Diet-induced weight loss alters hepatic glucocorticoid metabolism in type 2 diabetes mellitus

**DOI:** 10.1530/EJE-19-0901

**Published:** 2020-02-17

**Authors:** Andreas Stomby, Julia Otten, Mats Ryberg, Ruth Andrew, Brian R Walker, Tommy Olsson

**Affiliations:** 1 Department of Public Health and Clinical Medicine, Umeå University, Umeå, Sweden; 2 Region Jönköping County, Jönköping, Sweden; 3 Centre for Cardiovascular Science, Queen’s Medical Research Institute, University of Edinburgh, Edinburgh, UK; 4 Institute of Genetic Medicine, Newcastle University, Newcastle upon Tyne, UK

## Abstract

**Context:**

Altered tissue-specific glucocorticoid metabolism has been described in uncomplicated obesity and type 2 diabetes. We hypothesized that weight loss induced by diet and exercise, which has previously been shown to reverse abnormal cortisol metabolism in uncomplicated obesity, also normalizes cortisol metabolism in patients with type 2 diabetes.

**Objective:**

Test the effects of a diet intervention with added exercise on glucocorticoid metabolism.

**Design:**

Two groups followed a Paleolithic diet (PD) for 12 weeks with added 180 min of structured aerobic and resistance exercise per week in one randomized group (PDEX).

**Setting:**

Umeå University Hospital.

**Participants:**

Men and women with type 2 diabetes treated with lifestyle modification ± metformin were included. Twenty-eight participants (PD, *n* = 15; PDEX, *n* = 13) completed measurements of glucocorticoid metabolism.

**Main outcome measures:**

Changes in glucocorticoid metabolite levels in 24-h urine samples, expression of *HSD11B1* mRNA in s.c. adipose tissue and conversion of orally administered cortisone to cortisol measured in plasma. Body composition and insulin sensitivity were measured using a hyperinsulinemic-euglycemic clamp, and liver fat was measured by magnetic resonance spectroscopy.

**Results:**

Both groups lost weight and improved insulin sensitivity. Conversion of orally taken cortisone to plasma cortisol and the ratio of 5α-THF + 5β-THF/THE in urine increased in both groups.

**Conclusions:**

These interventions caused weight loss and improved insulin sensitivity with concomitant increases in the conversion of cortisone to cortisol, which is an estimate of hepatic *HSD11B1* activity. This suggests that dysregulation of liver glucocorticoid metabolism in these patients is a consequence rather than a cause of metabolic dysfunction.

## Introduction

Cushing’s syndrome, caused by overexposure of cortisol, is associated with abdominal adiposity, hypertension, dyslipidemia, insulin resistance and type 2 diabetes (1). This phenotype is strikingly similar to obesity and the metabolic syndrome. However, circulating cortisol levels are normal or slightly decreased in obese individuals (2), whereas studies suggest increased circulating cortisol levels in type 2 diabetes (3).

Circulating cortisol levels are regulated by the hypothalamic-pituitary-adrenal axis. Furthermore, cortisol exposure is regulated at the tissue level by a series of metabolic enzymes, with most attention paid to 11β hydroxysteroid dehydrogenase type 1 (HSD11B1) which converts inactive cortisone to active cortisol and HSD11B2 which converts cortisol to cortisone. Notably, HSD11B1 activity in adipose tissue is increased in individuals with obesity (4, 5) and hepatic *HSD11B1 *activity is decreased (4). However, among obese individuals with type 2 diabetes, hepatic *HSD11B1* activity is sustained (6, 7). This is important since *HSD11B1* has been highlighted as a potential drug target in type 2 diabetes (8).

The conversion of cortisone to cortisol by HSD11B1 is regulated by several factors related to obesity and type 2 diabetes (8). The increased expression of *HSD11B1* in s.c. adipose tissue has been associated with chronic inflammation (9), and treatment with anti-inflammatory salicylate decreases s.c. *HSD11B1* expression in obese patients (10). The decreased activity of *HSD11B1* in the liver among individuals with obesity has been suggested to be caused by chronic hyperinsulinemia, whereas the sustained hepatic *HSD11B1* activity among patients with type 2 diabetes may be caused by insulin resistance and a relative insulin deficiency (6, 11, 12). Furthermore, increased liver fat has been associated with increased excretion of glucocorticoid (GC) metabolites in urine and decreased hepatic *HSD11B1 *activity (13, 14). The association between anthropometric measurements, insulin sensitivity and liver fat with tissue-specific GC metabolism has not been tested among patients with type 2 diabetes.

Diet and exercise are fundamental components of the treatment of type 2 diabetes (15, 16). Among obese individuals without type 2 diabetes diet-induced weight loss has been associated with decreased excretion of GC metabolites in urine (17), decreased expression of *HSD11B1* in adipose tissue (18) but unaltered hepatic *HSD11B1* activity (17, 18). Notably, the effects of diet and exercise on tissue-specific GC metabolism have not been studied in patients with type 2 diabetes. Thus, it is currently unknown whether the benefits of weight loss and improved insulin sensitivity are associated with changes in tissue-specific GC metabolism.

The Paleolithic diet (PD) is considered to be a diet that humans have adapted to for thousands of years. Thus, the radical change in dietary intake during recent decades might explain the increased prevalence of obesity, type 2 diabetes and cardiovascular disease (19, 20). The PD causes a greater weight loss than a conventional diet according to Nordic Nutrition Recommendations among obese women without type 2 diabetes (21). Furthermore, the PD has been shown to improve insulin sensitivity and reduce hepatic lipid levels among patients with type 2 diabetes (22, 23). In general, the PD excludes the intake of dairy products, salt, refined sugar and grains, while the intake of vegetables, lean meat, fish and nuts is increased (21, 22).

Adding exercise training to dietary changes increases the beneficial effects on anthropometric measures as well as insulin sensitivity and glucose control (24). Furthermore, combining resistance and aerobic exercise is associated with larger improvements in glucose control than either modality alone, among patients with type 2 diabetes (16). The effect of adding exercise to dietary changes on tissue specific GC metabolism has not been tested in either individuals with obesity or patients with type 2 diabetes.

We aimed to (1.) test the association between anthropometric measurements, insulin sensitivity and liver fat with indices of tissue specific GC metabolism, (2.) test whether alterations in tissue specific GC metabolism can be reversed by weight-loss and associated improvements in insulin sensitivity and reduction in liver fat and (3.) test the additional effects of structured aerobic and resistance exercise to a PD on tissue specific GC metabolism in patients with type 2 diabetes. To achieve this we undertook analyses of secondary outcomes from an intervention trial for which the primary outcomes have already been published (22, 23).

## Subjects and methods

### Participants

Thirty-two weight-stable (<5% weight change last 6 months), overweight or obese (BMI 25–40 kg/m^2^) subjects diagnosed with type 2 diabetes within 10 years and treated with lifestyle modification ± metformin were included in this study. All participants were sedentary and had thus performed less than 30 min of moderately intense physical activity 5 days per week or resistance exercise less than once every other week during the last 6 months. Eligible men were 30–70 years old and women were included after menopause up to 70 years of age to avoid interference of menopause and the menstrual cycle on measurements of tissue-specific GC metabolism (25, 26). Exclusion criteria included use of antidiabetic drugs other than metformin, ischemic heart disease, stroke, blood pressure >160/100 mmHg, use of beta-blockers, being a smoker and macroalbuminuria.

All participants provided written informed consent before inclusion. The study was approved by the Regional Ethical Review Board, Umeå, Sweden and was conducted in accord with the Helsinki Declaration.

### Interventions

Participants were randomized to a Paleolithic diet alone (PD) or combined with a supervised exercise intervention (PDEX). Sixteen participants were randomized to each group, and one participant in the PD group and two in the PDEX did not complete the interventions. Furthermore, one participant in the PDEX did not provide repeated measurements on any tests of tissue-specific GC metabolism and was therefore excluded from the final analysis. The study was single-blind such that all staff collecting and analysing data were blind to group allocation.

The Paleolithic type diet included recommendations of a high intake of vegetables, fruit, lean meat, nuts, egg, fish and seafood. The intake of grains, sugar, salt, dairy products and refined fats was reduced (for details see (22)). Each group participated separately in five group sessions with a trained dietician at the Department of Food and Nutrition, Umeå University. They were given information, recipes and were shown how to cook a Paleolithic type diet. The first two meetings were held during the first two weeks after baseline measurements and thereafter once a month. The participants could contact the dietician by phone or email between sessions. Four-days of self-reported weighted food records at baseline and after 12 weeks were used to estimate diet adherence.

Before randomization, all participants met individually with a physician and were given recommendations of performing at least 30 min of moderately intense physical activity, for example, a brisk walk, each day in line with diabetes guidelines (27). In addition, 16 participants were randomized to a structured high intensity exercise intervention, added to the moderately intense physical activity recommendation, including three 1-h exercise sessions per week for 12 weeks. The exercise consisted of aerobic and resistance exercise (50% each) performed under supervision of an educated personal trainer at the Sports Medicine Unit, Umeå University. For details please refer 22. A standardized cardiopulmonary exercise test was used to measure peak oxygen uptake before and after the interventions.

### Body composition, anthropometric measures, liver fat and insulin sensitivity

Measurements were performed during a 3-week period before randomization and after the 12-week interventions. Weight was measured on a calibrated digital scale in light clothing, height was measured with a calibrated height-measuring gauge and waist circumference with a measuring tape midway between the iliac crest and lowest rib. Body composition was estimated with dual-energy X-ray absorptiometry (Lunar Prodigy X-ray Tube housing Assembly, Brand BX-1 L, Model 8743; GE Medical Systems, Madison, WI, USA). HbA1c was analyzed using routine clinical methods at the Department for Clinical Chemistry, Umeå University Hospital. Fasting glucose was analyzed from a capillary sample (HemoCue 201 RT; Radiometer Medical Aps, Brønshøj, Denmark). Cortisol binding globulin (CBG) was measured in serum using a competitive RIA (DIAsource ImmunoAssays, Louvain – la – Neuve, Belgium). The hyperinsulinemic-euglycemic clamp technique combined with (6,6-^2^H_2_) glucose infusion was used to assess peripheral and hepatic insulin sensitivity as previously described (23). Ten participants in the PD group and 12 participants in the PDEX group who provided measures of GC metabolism completed the hyperinsulinemic-euglycemic clamp at baseline and after 12 weeks. Liver fat content was measured using magnetic resonance spectroscopy (for details see Otten *et al.* (23)). Of the participants that provided measures of GC metabolism, liver fat content was measured in 14 participants from the PD group and 11 participants from the PDEX group.

### Excretion of glucocorticoid metabolites

The GC metabolites 5α-tetrahydrocortisol (5α-THF), 5β-tetrahydrocortisol (5β-THF), 5β-tetrahydrocortisone (5β-THE), free cortisol and cortisone were measured in 24-h urine samples that had been stored in −80°C for 1–3 years, using gas chromatography tandem mass spectrometry (28, 29). The ratios of these GC metabolites were then used as indices of *HSD11B1*, 5α-reductase and 5β-reductase activities as previously described (28, 29). In total, 12 participants from each group provided 24-h urine samples at baseline and 12 weeks.

### Cortisone conversion test

**Figure 1:**
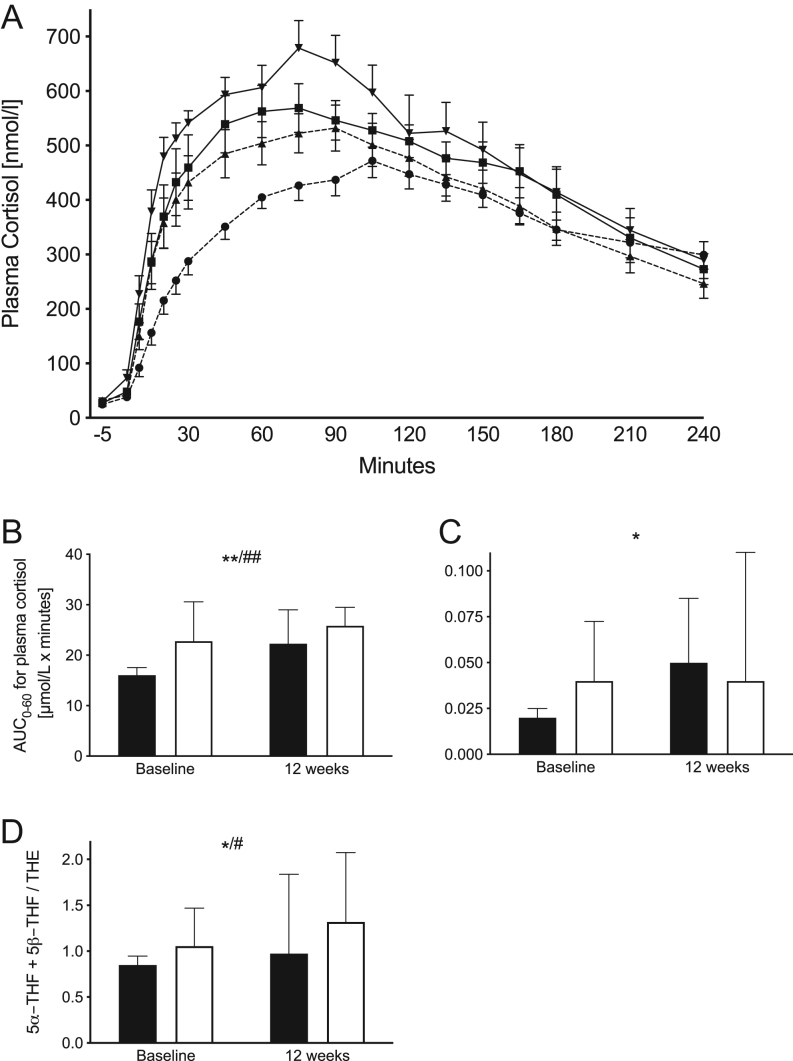
Results from the cortisone conversion test and excretion of GC metabolites to estimate hepatic *HSD11B1* activity. (A) Mean (s.e.m.) plasma cortisol levels during 4 h after orally administered cortisone acetate. Paleolithic type diet (PD) at baseline = dashed line with circles, 12 weeks = dashed line with triangles. Paleolithic type diet with structured exercise (PDEX) at baseline = line with squares, 12 weeks = line with triangles. (B) Median (IQR) area under the curve from 0 to 60 min (AUC0–60) during the cortisone conversion test (PD *n* = 14, PDEX *n* = 10). (C) Median (IQR) Ka constant calculated using curve fitting without compensation for lag (PD *n* = 9, PDEX *n* = 10). (D) The ratio of 5β-THF and 5β-THF to THE, an indirect measure of systemic *HSD11B1* activity. Black bars = PD, white bars = PDEX. *P < 0.05, **P < 0.01 for effect of time. ^#^*P* < 0.05, ^##^*P* < 0.01 for effect of group. There were no significant group × time interactions.

A cortisone conversion test was used to estimate hepatic *HSD11B1* activity. One milligram of Dexamethasone (Dexamethason, Galepharm, Küssnacht, Germany) was taken orally at 2300 h. At 0800 h the following day, a plasma sample was drawn and then participants took 25 mg of cortisone acetate orally (Cortison, Nycomed Pharma, Asker, Norway). Blood samples were drawn every 5 min for 0–30 min after the cortisone dose, every 15 minute for 30–180 min and every 13 min for 180–240 min (Fig. 1). Cortisol levels were analysed (Cobas E Cortisol regency kits, Cobas 6000, Roche Diagnostics) immediately at the Department of Clinical Chemistry, Umeå University Hospital. Two measures were used to estimate hepatic *HSD11B1* activity: (1.) the area under the curve for cortisol enrichment in plasma during the first hour after intake of cortisone, calculated with the trapezoidal method (AUC0–60) and (2.) a rate-of-appearance constant (Ka) calculated using a one compartment model of extravascular administration using curve fitting. The decision of compensating for lag due to slow gastric emptying was based upon visual estimation of the best curve fit (Kinetica Software, Thermo Fisher, Hemel Hempsted, UK). Fourteen participants in the PD group and 11 participants in the PDEX group participated in the cortisone conversion tests at baseline and after 12 weeks. Due to poor curve fitting at baseline, the Ka constant could not be estimated at all for three subjects, while it was excluded for two others in the PD group. One participant in the PDEX group had forgotten to take dexamethasone prior to the test and was therefore excluded.

### Expression of *HSD11B1* transcript in subcutaneous adipose tissue

Expression of *HSD11B1* was analysed in periumbilical s.c. adipose tissue. A needle biopsy was taken under local anesthesia, washed in saline and stored in −80°C for 1–2 years until analysis. Real-time quantitative PCR was used to analyze the abundance of *HSD11B1* mRNA. RNA was extracted using the RNeasy lipid tissue mini kit (Qiagen GmbH), and RNA concentrations were measured with a spectrophotometer (ND-1000, NanoDrop Technologies, Wilmington, DE, USA). Two micrograms of RNA were reverse transcribed using High Capacity cDNA RT kit (Applied Biosystems). Relative quantification real-time PCR was carried out using an ABI Prism 7000 Sequence Detection System or 7900HF Fast Real-time PCR (Applied Biosystems) using TaqMan Universal PCR Master Mix 2X (Applied Biosystems) and TaqMan Gene expression assays for the target gene *HSD11B1* (assay no. Hs01047362_m1, Applied Biosystems) and the endogenous control lipoprotein receptor-related protein 10 (LRP10, assay no Hs01047362_m1, Applied Biosystems). All reactions were performed in duplicate. Data were normalized against LRP10 and the comparative C_T_ method (ΔΔC_T_) used for relative quantification of *HSD11B1* gene expression. S.c. adipose tissue biopsies were taken from 11 participants in the PD group and 12 participants in the PDEX group at both time points.

### Statistical analysis

The Kruskal–Wallis test and visual inspection of Q-Q plots were used to assess normal distribution. The measures of tissue-specific GC metabolism that were not normally distributed were logarithmically transformed before statistical analysis. All data are presented as the median (interquartile range (IQR)). A two-way ANOVA was used to test for the effect of group, time and the time × group interaction. The effect of group tests for group differences at baseline or 12 weeks, effect of time tests for differences between baseline and 12 weeks in both groups and the time × group interaction tests for group differences in the effect from baseline to 12 weeks were observed. Expression of *HSD11B1* in s.c. adipose tissue and total excretion of urinary GC metabolites were significantly higher among men than women (*P* < 0.05) and therefore gender was included as a covariate for analyses including these measures. Spearman’s rank correlation coefficient was used to test correlations between measures of tissue-specific GC metabolism with BMI, waist circumference, body fat, fasting glucose, HbA1c, hepatic and peripheral insulin sensitivity and liver fat at baseline as well as changes in these measures during the interventions. In total, 48 correlational analyses were performed and the Bonferroni–Holm method was used as control for the family-wise error rate (30). A *P *value of < 0.05 was considered significant in all analyses.

## Results

### Participant characteristics

The main results from this intervention have been published previously (22, 23). This sub-study included 15 participants in the PD group and 13 participants from the PDEX group that provided any measure of GC metabolism (either 24-h urinary samples, s.c. adipose tissue biopsies or completed cortisone conversion tests) at both baseline and after 12 weeks. The median age was 61 years and the median diabetes duration was 3 years. All participants were treated with life-style modification and 68% were treated with metformin. The participants were relatively well controlled with respect to blood glucose levels with a median HbA1c of 55 mmol/mol at inclusion. None of these characteristics were different between groups except for fasting blood glucose, which was significantly higher in the PDEX group compared to the PD group at baseline (Table 1, *P* < 0.05 for effect of group).

**Table 1:** Baseline characteristics and intervention effects. Data are presented as medians (IQR).

	PD		PDEX	
	Baseline	12 weeks	Baseline	12 weeks
Gender (men/women)	10/5		8/5	
Age (years)	60 (11)		61 (8)	
Treated with metformin (yes/no)	10/5		9/4	
BMI (kg/m^2^)	31.4 (4.3)	28.9 (3.6)*	31.4 (6.1)	29.1 (6.7)*
Waist circumference (cm)	111 (12)	100 (18.4)*	107 (13)	99 (17.2)*
Body fat (%)	37.8 (9.2)	34.6 (9.4)	37.1 (9.8)	32.4 (11.5)
HbA1c (mmol/L)	53 (9)	42 (3)**	56 (16)	43 (5)**
Fasting glucose (mmol/L)	7.8 (1.5)^#^	6.3 (1.5)**	9.2 (3.4)^#^	7.1 (1.4)**
Suppression of hepatic glucose production (%)	89 (41)	108 (56)	108 (51)	126 (35)
Rate of glucose disappearance (mg/kg/min)	3.4 (1.3)	5.2 (3.2)*	4.0 (2.3)	5.4 (2.5)*
Liver fat (%)	22 (20)	5 (11)**	14 (19)	10 (22)**

*P < 0.05, **P < 0.01 for effect of time. ^#^*P* < 0.05, ^##^*P* < 0.01 for effect of group. There were no significant group × time interactions.

PD, Paleolithic diet; PDEX, Paleolithic diet with structured exercise.

### Diet and exercise adherence

As expected with given dietary recommendations, both groups reported a reduced intake of carbohydrates (PD: −24%; PDEX: −38%; effect of time *P* < 0.001) and saturated fatty acids (PD: −34%; PDEX: −31%, effect of time *P* < 0.001) and increased intake of protein (PD: 41%; PDEX: 39%; effect of time *P* < 0.001), monounsaturated (PD: 33%; PDEX: 83%; effect of time *P* < 0.001) and polyunsaturated fatty acids (PD: 60%; PDEX: 80%, effect of time *P* < 0.001). There were no significant effects of group or group × time interactions in the reported macronutrient intake. These data are presented in Table 2.

**Table 2:** Adherence to interventions. Data are presented as medians (IQR).

	PD		PDEX	
	Baseline	12 weeks	Baseline	12 weeks
Energy intake (kCal/day)	2022 (754)	1585 (894)**	1624 (871)	1316 (510)**
Carbohydrate intake (E%)	40 (10)	31 (16)***	45 (16)	28 (6)***
Protein intake (E%)	17 (5)	24 (10)***	18 (4)	25 (8)***
Total fat intake (E%)	39 (4)	42 (14)	32 (6)	44 (12)
Saturated fatty acid intake (E%)	15 (4)	10 (4)***	13 (4)	9 (2)***
Monounsaturated fatty acid intake (E%)	15 (3)	20 (10)***	12 (5)	22 (7)***
Polyunsaturated fatty acid intake (E%)	5 (2)	8 (3)***	5 (2)	9 (4)***
Peak oxygen uptake (ml/min/kg)	23.4 (6.4)	24.5 (7.4)**	22.8 (6.5)	28.7 (7.6)**

**P < 0.01, ***P < 0.001 for effect of time. There were no significant effects of group or group × time interactions.

E%, energy per cent; PD, Paleolithic diet; PDEX, Paleolithic diet with structured exercise.

All participants in the PDEX group completed >75% of all exercise sessions. In the sub-sample included in this study of GC metabolism, peak oxygen uptake increased in both groups (Table 2; PD: 8%; PDEX: 26%, effect of time *P* < 0.01) without a significant group × time interaction. For further details regarding exercise adherence see Otten *et al.* (22).

### Intervention effects on anthropometric measures, body composition and insulin sensitivity

Both interventions were associated with a reduction in BMI (PD: −8%; PDEX: −7%; effect of time *P* < 0.05) and waist circumference (PD: −10%; PDEX: −8%; effect of time *P* < 0.05). Fasting glucose levels (PD: −19%; PDEX: −33%; effect of time *P* < 0.01) and HbA1c (PD: −21%; PDEX: −23%; effect of time *P* < 0.01) decreased in both groups. The suppression of hepatic glucose production during the hyperinsulinemic-euglycemic clamp tended to increase in both groups (PD: 21; PDEX: 17%, effect of time *P* = 0.093), as did the rate of glucose disappearance (PD: 53%; PDEX: 35%, effect of time *P* < 0.05), indicating improved hepatic and peripheral insulin sensitivity. Liver fat decreased in both groups (effect of time *P* < 0.01) (23). These data are presented in Table 1. There were no significant group × time interactions indicating similar effects in both groups.

### Hepatic HSD11B1 activity

The rise in concentration of plasma cortisol after oral cortisone was significantly lower in the PD group at baseline (Fig. 1; AUC0–60 (PD: 15 712 nmol × h/L; PDEX: 22 888 nmol × h/L; effect of group *P* < 0.001), Ka (PD: 0.015; PDEX: 0.041; effect of group *P* = 0.13)). AUC0–60 and Ka increased after the intervention in both groups (Fig. 1, AUC0–60 effect of time *P* < 0.01; Ka effect of time *P* < 0.05). There were no significant group × time interactions indicating similar responses in both groups.

### Glucocorticoid metabolites in urine and expression of *HSD11B1* in adipose tissue

The ratio of 5α-THF+5β-THF/THE, reflecting systemic *HSD11B1* activity, increased in both groups following intervention (Fig. 1D and Table 3, effect of time *P* < 0.05). Otherwise, excretion of urinary GC metabolites and indices of 5α- and 5β-reductases were unaltered. Expression of *HSD11B1* in adipose tissue was unaltered in both groups. There were no significant group × time interactions.

**Table 3:** Measures of tissue-specific glucocorticoid metabolism. Data are presented as medians (IQR).

	PD		PDEX	
	Baseline	12 weeks	Baseline	12 weeks
5β-THF (mg/24 h)	3.3 (1.2)	2.9 (2.1)	3.1 (1.6)	3.3 (2.6)
5α-THF (mg/24 h)	3.2 (1.9)	3.7 (2.2)	3.5 (3.5)	4.3 (4.7)
THE (mg/24 h)	7.3 (4.5)	6.0 (7.1)	5.6 (2.8)	6.6 (4.2)
F (mg/24 h)	0.2 (0.1)	0.2 (0.2)	0.2 (0.1)	0.2 (0.1)
E (mg/24 h)	0.2 (0.1)	0.2 (0.2)	0.2 (0.1)	0.2 (0.1)
Total GC excretion (mg/24 h)	14.3 (7.3)	12.6 (11.9)	12.6 (6.1)	12.2 (12.3)
(5α-THF+5β-THF)/THE	0.85 (0.22)	0.98 (1.10)*	1.06 (0.57)	1.32 (1.13)*
5α-THF/F	14.06 (6.86)	15.73 (12.71)	16.25 (11.18)	22.8 (11.65)
5α-THF/5β-THF	0.98 (0.36)	1.19 (1.24)	0.93 (0.97)	1.30 (0.70)
THF/F	14.22 (8.06)	11.53 (7.20)	15.61 (6.35)	17.25 (13.69)
THE/E	35.70 (15.16)	27.04 (27.11)	29.92 (9.13)	30.15 (13.83)
F/E	1.02 (0.34)	1.06 (0.34)	1.04 (0.16)	0.92 (0.54)
*HSD11B1* expression (AU)	12.15 (9.59)	14.45 (7.47)	14.48 (9.02)	12.76 (9.33)
AUC0–60 (μmol × h/L)	15.7 (5.2)^##^	22.4 (11.3)**	22.8 (14.1)^##^	25.9 (4.4)**
Ka	0.015 (0.01)	0.052 (0.07)*	0.041 (0.06)	0.039 (0.08)*
CBG (nmol/L)	742 (146)^##^	726 (60)	856 (159)^##^	801 (114)

*P < 0.05, **P < 0.01 for effect of time. ^#^*P* < 0.05, ^##^*P* < 0.01 for effect of group. There were no significant group × time interactions.

5α-THF, 5α-tetrahydrocortisol; 5β-THF, 5β-tetrahydrocortisol; CBG, Cortisol binding globulin; E, cortisone; F, cortisol; PD, Paleolithic diet; PDEX, Paleolithic diet with structured exercise; THE, tetrahydrocortisone; total GC excretion, the sum of all measured glucocorticoid metabolites.

### Associations between tissue-specific glucocorticoid measures and anthropometric and biochemical measures

**Figure 2:**
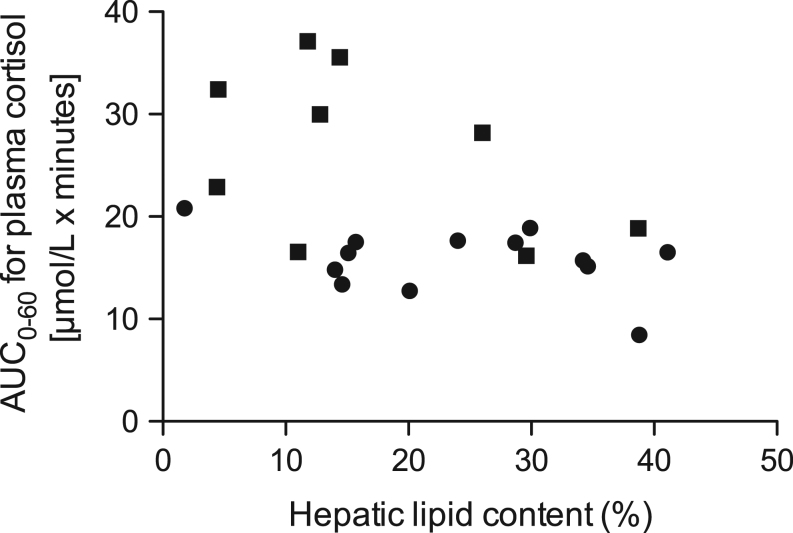
Area under the curve (AUC0–60) for plasma cortisol levels 60 min after a dose of orally taken cortisone was associated with hepatic lipid content at baseline. Dots represent the Paleolithic diet group and squares Paleolithic diet with exercise.

Before correction for multiple comparisons, a higher HbA1c level was associated with higher expression of *HSD11B1* in s.c. adipose tissue and increased excretion of urinary GCs (Table 4). Moreover, liver fat was associated with lower total excretion of GC metabolites and higher estimated hepatic *HSD11B1* activity measured by AUC0–60 for the rise in plasma cortisol concentration after oral cortisone (Fig. 2 and Table 4). After correction for multiple comparisons, the association between the HbA1c level and total excretion of urinary GCs remained significant.

**Table 4:** Associations between measures of tissue specific glucocorticoid metabolism and anthropometry, body fat, glucose control, insulin sensitivity and liver fat at baseline. The hyperinsulinemic–-euglycemic clamp was used to estimate hepatic insulin sensitivity as the suppression of hepatic glucose production and peripheral insulin sensitivity as the rate of glucose disappearance. Spearman rank correlations were used for cortisone conversion and partial correlations corrected for gender for HSD11B1 and urinary glucocorticoid metabolites.

	BMI	WC	Body fat	HbA1c	fP-glucose	HIS	PIS	Liver fat
Cortisone conversion (AUC_0__–60_)	−0.1	−0.35	0.13	0.002	0.35	−0.058	0.30	**−0.48**
*n*	25	25	25	25	24	19	19	**22**
*P*	0.6	0.09	0.5	0.9	0.09	0.8	0.2	**0.03**
Expression of *HSD11B1* in s.c. adipose tissue	0.028	0.063	0.19	**0.43**	0.35	−0.053	0.10	0.41
*n*	23	23	23	**23**	20	19	19	17
*P*	0.9	0.8	0.4	**0.04**	0.1	0.8	0.7	0.08
Total excretion of urinary glucocorticoid metabolites	−0.016	0.056	0.012	**0.63**	**0.60**	−0.32	−0.24	**0.63**
*n*	24	24	24	**24**	**23**	19	19	**21**
*P*	0.9	0.8	0.9	**0.001**	**0.003**	0.2	0.3	**0.003**

** WC, waist circumference; HIS, hepatic insulin sensitivity; PIS, peripheral insulin sensitivity.

There were no significant associations between anthropometry, body fat and hepatic or peripheral insulin sensitivity with other indices of GC metabolism (Table 4).

Despite these correlations at baseline, there were no significant correlations between changes in these measures during the interventions.

## Discussion

Our 12-week lifestyle interventions including a Paleolithic type diet with or without structured high-intensity exercise were associated with substantial weight loss and improvements in insulin sensitivity. Furthermore, the concentration of plasma cortisol after a single dose of cortisone, reflecting hepatic *HSD11B1* activity, as well as the ratio of 5α-THF+5β-THF)/THE, reflecting systemic *HSD11B1* activity, increased in both groups. There were no significant group differences in these effects.

The first aim with this study was to test the association between anthropometric measures, glucose control, insulin sensitivity and liver fat with tissue specific GC metabolism. Notably, liver fat was a predictor of both lower conversion of oral cortisone to plasma cortisol and increased excretion of urinary GC metabolites. This contrasts findings in obese individuals without type 2 diabetes in which liver fat was not associated with lower cortisone conversion, but increased excretion of 5β-reduced GC metabolites (13).

Poor glucose control, indicated by a high HbA1c, was associated with increased excretion of GC metabolites as well as increased expression of *HSD11B1* in s.c. adipose tissue. Notably, among women with impaired glucose tolerance, the mean glucose level during an oral glucose tolerance test has been associated with higher expression of s.c. *HSD11B1* as well (31). We did not find any associations between either hepatic or peripheral insulin sensitivity measured with the hyperinsulinemic-euglycemic clamp technique and any indices of tissue-specific GC metabolism. Previous studies have suggested that a relative insulin deficiency in type 2 diabetes may be the cause of sustained hepatic *HSD11B1* activity compared to obese individuals without type 2 diabetes (6, 12). Thus, we hypothesized that a higher degree of insulin resistance would be associated with increased hepatic *HSD11B1* activity. One reason for these unexpected results may be that all participants in this study had type 2 diabetes and that the effects of insulin resistance may be more evident in a group of patients with a larger variance in insulin resistance. The rather small study groups may also have limited the possibility to find significant associations between insulin resistance and HC metabolism.

Previous studies in non-diabetic obese individuals have found a clear association between BMI and waist circumference with excretion of GC metabolites in urine (4, 32, 33, 34). Surprisingly, this association was not evident in this group of type 2 diabetes patients. To summarize, our results suggest that liver fat, rather than the degree of insulin resistance or obesity, influences hepatic *HSD11B1* activity and GC metabolism. This should be further tested in larger samples of type 2 diabetes patients, since these associations did not remain significant after correction for multiple testing.

The second and third aim of the study was to test the effect of diet-induced weight loss with improved insulin sensitivity on tissue-specific GC metabolism and whether these effects are modified by intensive exercise. Previously, we have shown that a 2-year intervention with a PD associated with a 5–10% weight reduction increased the systemic activity of 5α-reductase and reduced the expression of *HSD11B1* in s.c. adipose tissue among obese women without type 2 diabetes (18). These results were not replicated in this shorter intervention among patients with type 2 diabetes. One reason for these discrepant results may be that an intervention longer than 12 weeks is needed to induce changes in GC metabolism. Furthermore, type 2 diabetes may also alter the effects of lifestyle interventions on tissue-specific GC metabolism compared to lifestyle changes among obese patients with normal insulin sensitivity.

The concentration of plasma cortisol after oral cortisone increased significantly after the interventions without a significant group difference. Numerically, the increase was greater in the PD compared with the PDEX group. However, since the cortisone conversion was significantly higher in the PDEX group compared to the PD group at baseline, probably caused by higher CBG levels (35) (Table 3), no firm conclusion can be drawn as to whether this tendency of group difference was due to a moderating effect of exercise training or the baseline difference *per se*. However, we have previously reported that exercise attenuated the loss of hepatic lipid content on a PD (23). Since higher liver fat was associated with lower cortisone conversion at baseline, it cannot be excluded that the addition of exercise also led to a reduced effect on hepatic *HSD11B1* activity based on the attenuated reduction of liver fat.

The rise in plasma cortisol concentration after oral cortisone depends both on the metabolic clearance rate of GCs, as well as the conversion of cortisone to cortisol by HSD11B1 in the liver (8). Excretion of urinary GC metabolites was unaltered in both groups suggesting an unaltered metabolic clearance rate. On the other hand, the ratio of cortisol to cortisone metabolites ((5αTHF + 5βTHF)/THE) in urine, considered to reflect systemic *HSD11B1* activity (28), increased in both groups. These findings support the hypothesis that the increased concentration of plasma cortisol after oral cortisone was caused by increased hepatic *HSD11B1* activity. Future studies could use tracer techniques to further elucidate these potential alterations in hepatic *HSD11B1* activity after weight loss (6).

The level of hepatic lipid content is a potential modifier of hepatic GC metabolism (13, 14, 36). It has been suggested that steatosis is associated with decreased conversion of cortisone to cortisol by hepatic *HSD11B1*, whereas steatohepatitis might lead to increased conversion (13, 14). In our study, a higher hepatic lipid content was associated with increased excretion of GC metabolites in urine and lower plasma cortisol after the cortisone conversion test at baseline. This may indicate that the reduction of hepatic lipid content during the interventions caused a reduced clearance rate of GCs and thereby increased concentration of plasma cortisol after the cortisone conversion test. However, it should be noted that there were no significant correlations between changes in these measures throughout the interventions and the ratio of cortisol to cortisone metabolites (5αTHF + 5βTHF/THE) in urine, reflecting systemic *HSD11B1* activity (28), was not correlated to hepatic lipid content. Moreover, a high-fat-low-carb diet with a carbohydrate intake as low as 4% has been suggested to increase systemic *HSD11B1* activity, measured with a tracer technique, independently of weight-loss in obese non-diabetic males (37). The participants in our study reported an intake of about 30% carbohydrates. Thus, the altered macronutrient composition could play a part in the increased hepatic *HSD11B1* activity, but is unlikely to be the sole contributor to these alterations.

Recently, metformin was shown to increase hepatic *HSD11B1* activity in subjects with type 2 diabetes, an effect likely linked to increased insulin sensitivity (38). We did not find any significant associations between estimated hepatic *HSD11B1* and neither peripheral nor hepatic insulin sensitivity measured with the hyperinsulinemic-euglycemic clamp technique. The cortisone conversion test is a less specific and sensitive test to estimate the hepatic *HSD11B1* activity compared with the more recently developed tracer techniques (5, 38). This may, at least in part, explain these different findings.

Using indirect measures to estimate tissue-specific GC metabolism has limitations (8). As discussed, it is not always straightforward to infer mechanistic tissue-specific changes in GC metabolism by using these indirect measurements. Using tracer techniques and hepatic vein catheterization, would ultimately provide more specific and sensitive estimates of hepatic *HSD11B1 *activity (5). Our sample size was fairly small with a limited power to detect significant effects which may have affected some of our results. Some participants who took part in the interventions did not take part in measures of tissue-specific GC metabolism at both baseline and 12 weeks and were therefore excluded. The specific reason for not taking part was not documented. The PDEX group reported a non-significant lower total intake of calories at baseline and after 12 weeks than the PD group. However, all participants reported stable weight for the last 6 months, and the reduction in weight and waist circumference during the interventions were similar. This difference is likely an effect of underreported calorie intake rather than actual differences, which is common with self-reported food intake. Those treated with metformin had a significantly higher BMI, HbA1c and more insulin resistance (data not shown) compared to those treated with life-style modification. This limited the possibility to test the effects of metformin, which could have been relevant with respect to previous studies (38). There was a large variance in hepatic lipid content and hepatic insulin sensitivity within and between groups at baseline. Although these differences were not statistically significant, they may have influenced the effects of the interventions. We tested several associations between measures of GC metabolism and anthropometry, insulin sensitivity and liver fat. These tests were based on hypotheses from previous studies, but after correction for multiple testing, several associations did not remain significant. All women included were postmenopausal, so we cannot say if other associations/responses are present in premenopausal women, as suggested by earlier studies (26).

To conclude, intervention with a PD was associated with increased cortisone conversion, reflecting increased hepatic HSD11B1 activity. Adding exercise to a PD did not have a significant impact on these effects.

## Declaration of interest

The authors declare that there is no conflict of interest that could be perceived as prejudicing the impartiality of this study.

## Funding

This work was funded by the County Council of Västerbotten (VLL-460481), the Swedish Heart and Lung Foundation (20120450), King Gustav V and Queen Victoria’s foundation and the Swedish Diabetes Research Foundation (2014-096). B R W and R A are supported by grants from British Heart Foundation and Wellcome Trust.
